# Our New York Letter

**Published:** 1890-01

**Authors:** M. B. Hutchins

**Affiliations:** New York


### Correspondence.

### OUR NEW YORK LETTER.

New York, November, 1889.

A woman now in the hospital has an immense tumor in right breast, and on surface a fungating growth, cauliflower-like in appearance. There are numerous hard nodules in left breast and in skin of chest. Dense lymphatic nodules in each axilla. Patient is only thirty-six years old. Age of patient, rapid growth of disease and fungating appearance of part described, suggested sarcoma to the surgeon, but hard infiltrates could only occur in carcinoma, or true cancer. While still doubtful of the diagnosis, the doctor noticed a number of superficial scars on chest and shoulders, possibly suggestive of hereditary syphilis. Mentioned the fact that Esmarch had, after long study of the subject, reached the conclusion that ninety per cent. of sarcoma cases occurred in those who had suffered from hereditary syphilis. However, the diagnosis is carcinoma; and the doubt only served to bring out the interesting theory of Esmarch. Another case, in every way suggestive of sarcoma, was proven by the microscope to be carcinoma. In New York sarcoma, other than sarcoma cutis, is very rare.

A prominent dermatologist mentions an uncommon localization in an attack of gout of which he himself suffered. The pain and swelling were located just at beginning of the tendo-achillis and in annular ligament of ankle. Attack followed drinking a half bottle of claret, to which the doctor was unaccustomed. Antipyrin and acetate of potassium relieved the trouble, on theory of rheumatic relationship.

Dr. G. H. Fox is a believer in "diet," as important in treatment of skin diseases. He thinks a vegetable diet better than one of meat, but has confined a patient to beefsteak alone. His theory, however, is that a change of digestive habit is good for the stomach, and, reflexly, for the patient, this change of habit probably deserving to be classed with change of scene and habit of life as beneficial agents. A "skin specialist" mentions having seen a case of syphilis in which an outbreak of the tubercular syphilide occurred upon the borders of scars left by former lesions of the disease.

Hydroxylamin, which seemed to promise so much, has apparently fallen among forgotten things. It belongs to the "reducing agents," new ones of which are constantly being brought out by dermatologists. As such remedies were supposed to be indicated in psoriasis—chronic form—each addition to the list was given a trial. Blaschko, at a meeting of the Berlin Dermatological Society, said that he had experimented with the "reducing agents," and had reached the conclusion that the good effect in psoriasis was not through this action, but all substances which produced rapid desquamation could cure the disease. He, and others, had seen cases get well after the desquamation in an attack of scarlatina. This view clears up the theory, and may lighten the practice, of treatment for psoriasis.

A peculiar case of palmar eczema (?) began—a year ago with heat and burning in palm of left hand; then small, dark red macules appeared along proximal edge of palm and at base of thumb, these lesions going on to desquamation. Disease progressed by formation of new patches adjacent to primary, which in turn became pale and desquamated. Right hand became affected a month later, process following similar course. Finally entire palms and palmar surfaces of fingers affected, deep red and "burning." Disease encroaches only slightly upon palmar borders, but the sides of fingers were affected, and the last phalanx practically surrounded over matrix of nail. Epidermis thickened and rapidly thrown off. Nails grew faster, appeared brittle and were somewhat thickened by "sub-ungual" accumulation of epidermis. At beginning of treatment in the hospital the condition was as follows: Surfaces described of deep red color, thickening slight; formation of numerous small, rather adherent scales. Painful to touch, no loss of fluid, no "weeping." On flexor surfaces of forearms a number of large papules, pale red in color. Disease did not look like "specific," nor was there any history of the "infectious" disease. Patient had used pearline in house-work previous to and after appearance of disease.

TREATMENT.—℞: Ac. salicyl., gr. x; magnesiæ carb., amyli, aa. gr. xv; ungt. zinc. ox., ℥i. M. Sig.: Apply to hands and arms. Eruption on arms rapidly declined. No effect on palms. After a thorough trial of above, ℞: Ungt. picis et zinc. ox., ungt. zinc. ox., aa. M., applied to palms, and made them worse. Then lotio ichthyol, three per cent., was used, applied on cloths. Gave relief for two days, then the affected surfaces again became deep red, pain intense, and a feeling as of subcutaneous effusion; giving appearance of erysipelas. Immersion of hands in hot water gave no relief; warm water slightly soothing. Then the palms and fingers were sprayed for about two hours with ether. This reduced tense appearance of skin, and finally patient became comfortable. Hands now dressed with a lotion of calamine, zinc oxide, lime water, etc. The appearance of the disease and its resistance to treatment suggest the possibility of a local pityriasis rubra.

What might be called a a new method of "ring-firing" in the treatment of erysipelas is that of scarifying the skin in advance of progressing edge—thus XXXXXX. A believer in micro-organisms says it is aseptic treatment—a kind of ditching off, as it were (like they check the creeping plague of grasshoppers in the East). This method of treating erysipelas is said to produce a quick cure.

The following history of a case of lupus vulgaris gives an idea of the obstinacy of the trouble, and the treatment includes nearly every (old and new) method of managing the disease:

March 2d, 1888.—G. C., aged twenty-six years. Disease began eight years ago, at which time patient had naso-pharyngeal catarrh. Bluish "spot" appeared near ala of nose; upper lip became affected with numerous "spots," discharging whitish matter. Nose and adjacent parts of cheeks next implicated. Upon patient's entrance, disease extended over upper lip, nose and cheeks; right most affected. Similar disease apparently in pharynx and soft palate.

TREATMENT.—Had previously taken Hood's sarsaparilla and cathartics. Next used ointment of carbolic acid, and the solid stick (?) nitrate of silver. After an intermission of treatment electrolysis used. After ten days in country branch of the Skin and Cancer Hospital, patient was etherized and "tubercles" of right side of face scraped out with curette; curetting of left side four days later. Dressing consisted of ungt. resorcini (five per cent.) Improved.

May 1st.—Began taking pil. phosphori, gr. $\frac{1}{100}$ t. i. d.

May 9th.—Nitrate stick used to "bore out" tubercles—many still being present. Scarifying (Vidal's method) employed in places, curetting continued in others. Less redness then present and fewer tubercles. Face dressed with, ℞: resorcini, gr. l; zinc ox., ʒ ss.; ac. carb., gr. v; ungt. ox., ℥i. M. No record of treatment of pharynx.

Six months after entering hospital tubercles of disease few and hard to discover, but on Septtember 17 two small nodules felt on right cheek, at lower border of diseased area, and apparently a few characteristic points on end of nose.

October 4th.—Discharged.

November 8th.—Re-admitted. No treatment in interval. New tubercles present at various points in cicatricial area. Former treatment continued. Improved. While taking phosphorus, gr. ¼ *per diem*, mentioned state of "nervousness," interfering with sleep, which disappeared to some extent after reduction of dose. In December lotion of nitrate of silver ʒi—℥i used after scarification, and "solid stick" in some of diseased points. Meanwhile the regular resorcin and carbolic ointment was employed in varying strength. Phosphorus pills now discontinued.

In January a continued recurrence of lupus nodules and tubercles. Patient then away for two months.

March.—Returned in *statu quo.* ℞: Resorcin, gr. xl; ungt hydrarg. ammoniat., ʒ ii ; ungt. aq. rosæ, ad. ℥ i. M. Applied to face, and tonics given internally.

March 12th.—℞ : Hydrarg, iodid. rub. gr. xx; lanolin ʒii; ungt. aq. ros. ad. ℥i. M. used on right side of face, former on left. Pain caused by each; swelling by the ointment on left side.

March 19th.—"Campho-phenique" added to last ointment, seemingly relieving some of the pain.

March 25th.—Face inflamed, skin exfoliating, but a number of tubercles still visible. Ointments applied for two hours and followed by starch poultice for a few hours; then washed off and ℞: ac. carb. gr. iv; ungt. zinc. ox. ℥i. M. applied, giving some relief to acute symptoms.

April 9th.—℞ : Ichthyol ʒi; ac. chrysophanici ʒss ac. salicyl. ʒi; zn. ox. ʒii; bals. canadensis ad. ℥ii. M. This made as physical properties required and substituted for previous applications.

April 12th.—Entered City Hospital. For three days used plaster containing ichthyol, chrysarobin, ac. salicylic and oxide of zinc. (This is the plaster the good effect of which, in a case of lapus, was mentioned in a former letter. That case is now nearly cured, but the plaster was soon displaced by Vidal's method of linear scarification.) Plaster was followed by a paste containing same ingredients. No effect. After ten days returns to plaster, which had some effect in causing "points" to suppurate. Gradually lost effect, and ℞: hydrarg. ox. rub. gr. xxx; resorcini gr. xx; lanolini ʒii; ungt. simp. ad. ℥i. No action even after increase of two first ingredients.

May 25th.—Begins Thompson's sol. phosphorus gtt. xv, t. i. d. increasing gtt. ii, *per diem.* Curetted, and ointment well rubbed in.

June 21st.—Ungt. hydroxylamini three per cent. substituted. Face swells. Reduce to two p. c. Some effect on tubercles; three p. c. resumed. Then a modification of Unna's treatment, with strong solution of bichloride, was employed. Wooden tooth-pick dipped in sol. and bored into tubercles; carbolic dressing. Effect not striking. Goes home.

October 22d.—Returns with limited number of tubercles recurred, mainly on lower border of patch on right cheek. Using emplastrum hydronaphtholis, with only superficial effect. Resumed Thompson's solution of phosphorus, getting now about ℥ii *per diem*. Ordered ℞ :hydronaphtholis, amyli aa. M. To be applied as a paste.

A review of the history as well as observation of the case for over a year showed that curetting and scarification have acted better than any applications, with, perhaps, a difference in favor of the linear scarification. Contrary to the theory of circulatory absorption of the tubercle bacillus through curetted or incised skin, there is no evidence of conveyance of the disease to other parts, and the patient is in good general health.

Subcutaneous injections of mercurials in syphilis are scarcely practiced here. The majority of authorities abroad have concluded that the disadvantages of this method overweigh the advantages. Sometime ago a woman was brought into Bellevue Hospital with diffuse yellowish brown pigmentation of the whole skin, only a few white cicatricial looking lines being visible. Quite a number who saw the case were misled by the statement that the pigmentation had taken place in *two days*. Finally Dr. Piffard saw the case and said appearance resulted from "*age and vermin*."

M. B. HUTCHINS, M. D.

